# Morphological Characterization of a New and Easily Recognizable Nuclear Male Sterile Mutant of Sorghum (*Sorghum bicolor*)

**DOI:** 10.1371/journal.pone.0165195

**Published:** 2017-01-04

**Authors:** Zhanguo Xin, Jian Huang, Ashley R. Smith, Junping Chen, John Burke, Scott E. Sattler, Dazhong Zhao

**Affiliations:** 1 Plant Stress and Germplasm Development Unit, USDA-ARS, Lubbock, Texas, United States of America; 2 Department of Biological Sciences, University of Wisconsin-Milwaukee, Milwaukee, Wisconsin, United States of America; 3 USDA-ARS-PA-Grain, Forage, & Bioenergy Res. Unit, 251 Filley Hall/Food Ind. Complex, Lincoln, Nebraska, United States of America; Henan Agricultural University, CHINA

## Abstract

Sorghum (*Sorghum bicolor* L. Moench) is one of the most important grain crops in the world. The nuclear male sterility (NMS) trait, which is caused by mutations on the nuclear gene, is valuable for hybrid breeding and genetic studies. Several NMS mutants have been reported previously, but none of them were well characterized. Here, we present our detailed morphological characterization of a new and easily recognizable NMS sorghum mutant *male sterile 8* (*ms8*) isolated from an elite inbred BTx623 mutagenized by ethyl methane sulfonate (EMS). Our results show that the *ms8* mutant phenotype was caused by a mutation on a single recessive nuclear gene that is different from all available NMS loci reported in sorghum. In fertile sorghum plants, yellow anthers appeared first during anthesis, while in the *ms8* mutant, white hairy stigma emerged first and only small white anthers were observed, making *ms8* plants easily recognizable when flowering. The ovary development and seed production after manual pollination are normal in the *ms8* mutant, indicating it is female fertile and male sterile only. We found that *ms8* anthers did not produce pollen grains. Further analysis revealed that *ms8* anthers were defective in tapetum development, which led to the arrest of pollen formation. As a stable male sterile mutant across different environments, greenhouses, and fields in different locations, the *ms8* mutant could be a useful breeding tool. Moreover, *ms8* might be an important for elucidating male gametophyte development in sorghum and other plants.

## Introduction

Sorghum (*Sorghum bicolor* L. Moench) is the fifth most important grain crops in the world, providing food of subsistence to over 500 million people in Africa and South East Asian [1]. As a C4 crop with excellent tolerance to drought and high temperature stresses as well as adaptation to marginal soils, sorghum is becoming increasingly important as a promising bioenergy crop for sugar, biomass, and biofuel production [2, 3]. With a moderate sequenced diploid genome (~730 Mb), sorghum is also an emerging model for highly productive C4 crops [4, 5].

Male sterile mutants are key tools for hybrid breeding. Male sterility in flowering plants is often attributed to a failure in pollen production or shedding due to defective anther development or dehiscence, while the development of female floral organs is normal [6]. There are two types of male sterility: cytoplasmic male sterility (CMS) and nuclear male sterility (NMS) or genic male sterility. CMS, which is maternally inherited, is caused by incompatibility of the cytoplasmic genome with the nuclear genome [6]. The CMS must be maintained by pollination with a companion line that has a nearly identical nuclear genome but a compatible cytoplasm. Genetic defect in the nuclear genome results in NMS, which is usually segregated as a recessive trait in the self-pollinated F2 offspring.

Male sterility has played a major role in production of hybrid seeds in large scale to exploit the phenomenon of heterosis. At present, CMS is predominantly used for hybrid production in crops. In the CMS breeding system, three lines are required for hybrid seed production [7]. The female parent (A line) is completely male sterile. A maintainer line (B line) is needed to pollinate the A line for maintaining the absolute male sterility of the A line. During hybrid seed production, a male parent (R line) is required to provide pollen to the A line, restoring the fertility of the F1 plants by complementing the cytoplasmic defect of the A line with dominant restoring factors in the nuclear genome [8]. Simultaneous development of all three lines in breeding hybrids is very complicated and expensive. Moreover, strict requirements for A, B, and R lines severely limit the use of germplasm accessions for generating all possible hybrid vigor. In rice, two-line hybrid breeding systems using conditional nuclear male-sterility have been explored to simplify the hybrid seed production procedure and to expand the possibilities of making hybrids between accessions that are not possible with the three-line breeding system [9–11]. In the two-line breeding system, a conditional male sterile line is used as the male sterile line under restrictive conditions and the self-maintainer line under permissible conditions [9]. Any other lines that do not contain the same conditional male sterile mutation can serve as the “restorer” line. Thus, there is no limitation to the use of germplasm accessions to make hybrids as long as the heterosis between two lines is acceptable.

In sorghum, CMS is exclusively used for hybrid breeding [7, 12]. Although several types of cytoplasmic male sterile lines are available, A1 cytoplasm is mainly used in commercial hybrid production [8, 13]. The A1 cytoplasmic homogeneity may predispose sorghum hybrids to the devastation of diseases as what happened to A-cytoplasmic maize hybrids in 1970s [14]. Because NMS lines have normal cytoplasm, they may have the potential to reduce catastrophic yield losses if they can be configured to make hybrids either through conditional or inducible male sterility. Eight different NMS lines have been reported previously in sorghum [15]. Unfortunately, several NMS lines are not available now. Due to the diligent effort of Jeffrey Pedersen, five of the reported NMS lines, i.e. *ms1*, *ms2*, *ms3*, *ms7*, and *msal*, have been preserved and introduced into different genetic backgrounds [16]. However, it is difficult to distinguish these mutants from the wild-type plants, because their floret structures, including the anther, are similar to that of wild type. Furthermore, none of these NMS mutants were morphologically and genetically characterized.

Here, we show our morphological studies on a new easily recognizable NMS mutant, *male sterile 8* (*ms8*), which was isolated from an ethyl methane sulfonate (EMS)-mutagenized mutant population in sorghum inbred line BTx623. Compared with wild type and the previously reported NMS lines, *ms8* can be easily recognized at onset of anthesis, because its anthers, which emerged later than stigmas, are small and white. Genetic analysis supports that the male sterile phenotype is mediated by a single recessive nuclear gene mutation. Complementation experiments show that the *ms8* mutant is a new NMS mutant that is different from any of *ms* lines currently available. Further examinations demonstrate that the *ms8* mutant is normal in ovary development, but it is defective in tapetum development, which causes no production of pollen grains. As an easily recognizable NMS, *ms8* may be a useful tool in applications of sorghum breeding.

## Materials and Methods

### Generation of Sorghum Mutant Library

The sorghum [*Sorghum bicolor* (L.) Moench] inbred line BTx623 seeds were obtained from the National Germplasm Resources Information Network of USDA-ARS (http://www.ars-grin.gov/). After six generations of purification through single seed descent, the BTx623 seeds were mutagenized through treatment with EMS at concentrations ranging from 0.1 to 0.3% (v/v) as described previously [17]. The treated seeds were thoroughly washed in about 400 ml of tap water for five hours at ambient temperature, with changing of the wash water every 30 min. Then the mutagenized seeds were air-dried and prepared for planting.

### Field Planting and Management

The sorghum mutant library was planted annually on the Research Farm of the Plant Stress and Germplasm Development Research Unit, USDA-ARS, Lubbock, Texas, USA (latitude 33° 35’ N, longitude 101° 53’ W, and altitude 958 m). The soil type is an Amarillo fine sandy loam (fine-loamy, mixed, superactive thermic Aridic Paleustalfs). Before planting, a mixture of bulk ammonium sulfate and mono ammonium phosphate was applied to the field, calculated to achieve levels of 65 kg nitrogen and 27 kg phosphorous per hectare. The plot size is 4.67-m long with 1.02-m row spacing. Sorghum seeds were planted at 80 per row at a depth of 3 cm using a John Deere MaxEmerge Planter. The plots were watered from underground drip lines as needed to maintain sufficient soil moisture.

### Screening of the NMS Mutant

In 2010, a panicle with no extruded anther was observed from a plant in plot 3049, in which the mutant line 25M2-1075 was planted. The main shoot of the plant that bore the sterile panicle was cut to stimulate tiller growing. Four tillers were developed later. One tiller was left open. Three tillers were bagged before heading. One of the three bagged tillers was pollinated with BTx623 wild-type pollen when the stigma had extruded from approximately 50% of the sessile spikelets. The other two panicles were continually bagged until harvesting. Neither of the two continually bagged tillers set any seed. However, both the open pollinated panicle and the manually pollinated panicle set seeds. The F1 plants from both open-pollinated and manually pollinated panicles were completely fertile, suggesting the male sterility mutation was recessive. The F2 plants derived from the manually-pollinated F1 progeny segregated 9 male sterile to 31 fertile, a ratio of approximately 1 to 3. Because of the easiness to identify the male sterility phenotype, we continued to backcross *ms8* to BTx623 to develop a near isogenic line to serve as a convenient tool for backcrossing other mutants isolated from the mutant library.

### Examination of Female Fertility

After panicles were emerged, the *ms8* mutant plants were determined by the anther phenotype. The top parts of BTx623 and *ms8* panicles were cut and bagged. One day later, the cut panicles were manually pollinated by the BTx623 pollen. Ovaries were dissected out from panicles before as well as 2 and 3 days after pollination. Ovaries were observed and imaged with the Olympus SZX7 dissection microscope equipped with an Olympus DP 70 digital camera (Olympus, Center Valley, PA, USA).

### Pollen Staining and Anther Sectioning

Alexander staining was used to determine pollen viability as described previously [18, 19]. Briefly, anthers just before anther dehiscence were dissected out and fixed 24 h in the fixative (methanol, 60mL; chloroform, 30mL; distilled water, 20 mL; picric acid, 1 g; and HgCl_2_, 1 g). Anthers were transferred through 70%, 50%, and 30% ethanol followed by distilled water (1 h in each change) and incubated in the staining buffer (ethanol 95%, 10 ml; malachite green, 10 mg; acid fuchsin, 50 mg; orange G, 5 mg; phenol, 5 g; glacial acetic acid, 2 ml; glycerol, 25 ml; and distilled water 50 ml) at 50°C for 48 h. Anthers were mounted on the glass slide for observation.

Semi-thin sectioning was carried out as described in our previous studies [18, 20]. Sorghum spikelets were fixed in the fixative (2.5% glutaraldehyde, 0.1M HEPES, 0.02% Triton X-100, pH7.2) overnight at room temperature. Samples were washed three times (30 min each) in the wash buffer (0.1M HEPES, 0.02% Triton X-100, pH7.2) and then post fixed in 1% OsO4 overnight at room temperature. Samples were dehydrated through an acetone series (10% increments, 1 h each change) and infiltrated in 20%, 40%, 60%, and 80% of low viscosity Spurr’s resin (3 h each change). Samples were then transferred into 100% Spurr’s resin three times (24 h each change) and embedded in 100% Spurr’s resin. Samples were finally polymerized at 60°C overnight. Semi-thin (0.5 μm) sections were performed using an Ultracut E ultramicrotome (Reichert-Jung) and were stained with 0.05% of Toluidine Blue O. Images of pollen staining and anther semi-thin sections were photographed with an Olympus BX51 microscope equipped with an Olympus DP 70 digital camera (Olympus, Center Valley, PA, USA).

## Results

### The *ms8* Mutant Is an Easily Recognizable NMS Mutant

**Fig 1:**
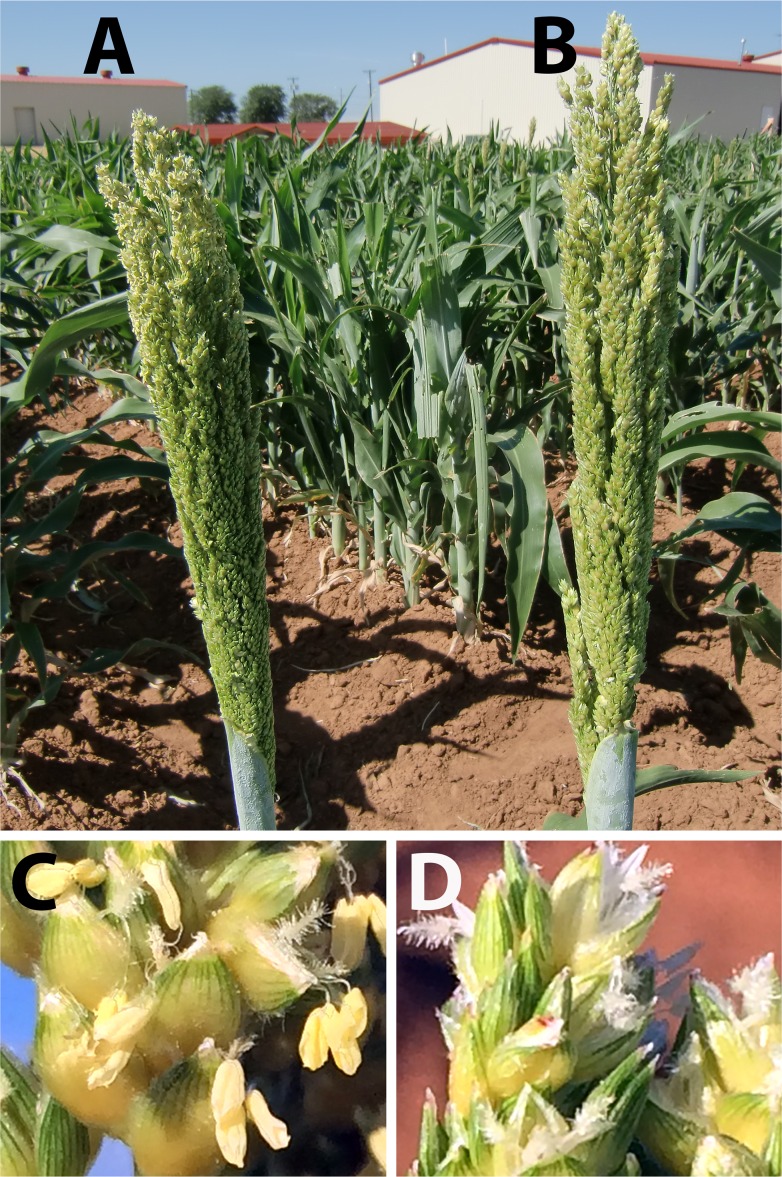
Comparison of BTx623 wild-type panicle and spikelets with that of the *ms8* mutant. **(A)** Fresh yellow anthers were extruded from sessile spikelets of the BTx623 wild-type panicle at the anthesis stage. **(B)** Anthers were small and white in *ms8* spikelets at the anthesis stage; thus they were nearly invisible in the panicle. **(C)** Yellow anthers are shown in BTx623 spikelets at the anthesis stage. **(D)** White small anthers and hairy stigmas were observed at the anthesis stage.

In the wild-type BTx623, yellow anthers appeared earlier than stigmas in all sessile spikelets during anthesis (Fig 1A and 1C). Conversely, anthers in the *ms8* mutant were small and white (Fig 1B and 1D). Furthermore, white hairy stigmas emerged before anthers in the *ms8* mutant. Thus, the *ms8* mutant can be easily recognized via observing clearly visible white anthers and stigmas in all sessile florets at the beginning of anthesis.

**Fig 2:**
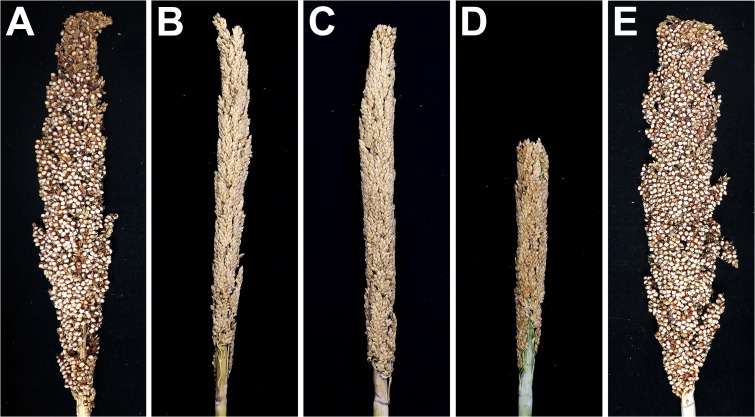
*ms8* is a stable male sterile mutant. **(A)** A self-pollinated BTx623 panicle showing the normal seed set. **(B-D)** *ms8* panicles bagged before anthesis showing no seed set from fields in Lubbock, TX (B) and Puerto Rico (C), as well as under the greenhouse condition (D). **(E)** A manually pollinated *ms8* panicle showing the normal seed set.

Compared with the wild type (Fig 2A), no seeds were produced in the *ms8* panicle (Fig 2B). The seed production of *ms8* mutant plants was fully recovered when pollinated with BTx623 pollen grains (Fig 2E), indicating that *ms8* is a male sterile mutant. To eliminate the effects of other unlinked mutations, we backcrossed *ms8* to the wild-type BTx623 for six generations. The *ms8* mutant plants were never observed to produce any seeds in the absence of a pollen source over the last several field seasons in Lubbock, TX, USA (Fig 2B) and Puerto Rico (Fig 2C), as well as under greenhouse conditions (Fig 2D). Our results demonstrated that the *ms8* male sterility phenotype can be easily recognized and stable. The backcrossed *ms8* can be routinely used as a near isogenic line of BTx623 to backcross other plants to avoid hand emasculation or plastic-bag crosses [7].

### The Female Fertility Is Normal in the *ms8* Mutant

**Fig 3:**
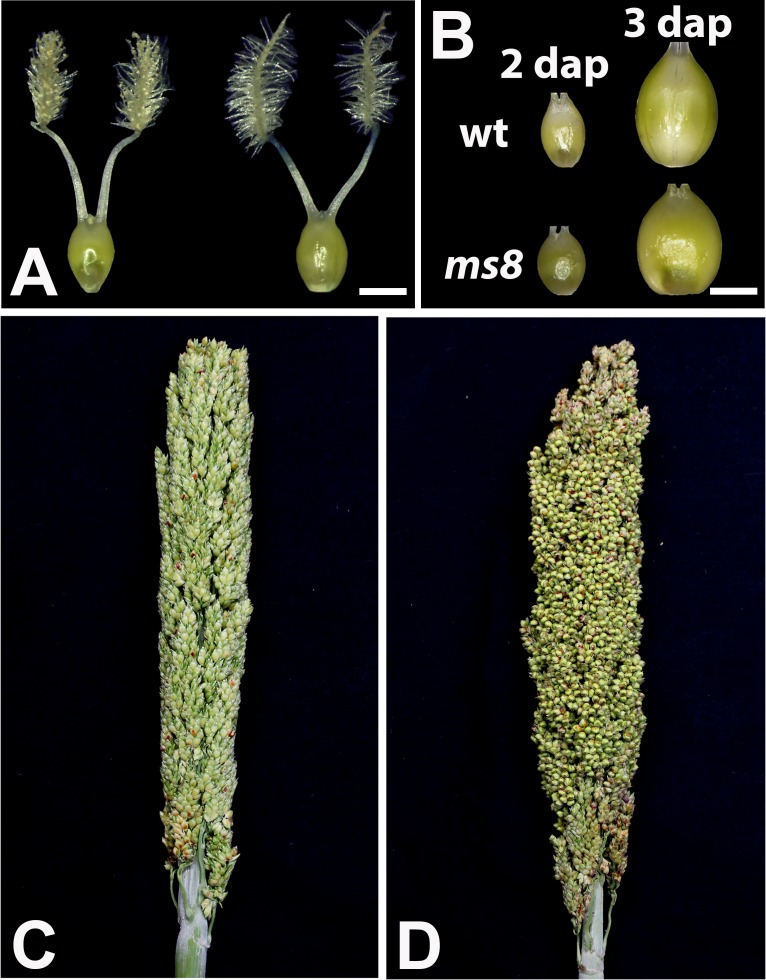
The female fertility is normal in the *ms8* mutant. **(A)** The BTx623 wild-type ovary (left) was the same as that of the *ms8* mutant (right) without manual pollination. **(B)** There was no difference of ovary development between BTx623 and the *ms8* mutant after manual pollination. dap: days after pollination. Bars = 1 mm in A and B. **(C)** An *ms8* panicle bagged before anthesis showing no developing seeds. **(D)** Seeds are being normally developed in a manually pollinated *ms8* panicle.

To examine whether female organs are affected by the *ms8* mutation, we examined ovaries before and after manual pollination in *ms8* plants. We found that there was no difference in ovary size and appearance before pollination (Fig 3A). After manual pollination, ovaries in both wild-type and *ms8* mutant plants developed similarly (Fig 3B). Without pollination, the *ms8* panicle had no developing seeds (Fig 3C). However, after the manual pollination, a full set of normally developing seeds were observed (Fig 3D). Therefore, our results suggested that the *ms8* mutation only affected the male sterility but had no effect on the female fertility.

### *ms8* Is a Novel Male Sterile Locus Distinct From All NMS Lines Currently Available

Our results provided several lines of evidence to support that *ms8* is a recessive mutation in a nuclear encoded gene. First, the F1 plants between a cross of the *ms8* mutant and the wild-type BTx623 were completely fertile (Table 1), suggesting that *ms8* is a recessive mutation. Second, the male sterility was segregated as a single recessive nuclear gene mutation during the subsequent backcrosses to the wild-type BTx623. Third, the F2 plants after six backcrosses segregated as 108 male sterile and 320 fertile (Table 1). Statistical analysis indicates that the 1:3 (mutant:wild type) segregation ratio can be accepted according to the X^2^ test (Table 1), suggesting that the *ms8* recessive mutation occurred in a single nuclear gene. Furthermore, to increase the frequency of male sterile plants that can be used for backcrossing, we crossed the *ms8* heterozygous plants (fertile) with the *ms8* homozygous mutant plants (male sterile). Fifty plots (4.5 m × 1 m) of resulting F1 seeds were planted in the winter nursery in Puerto Rico from November 24, 2015 to 18 March 2016. Three plots were examined for male sterile and fertile plants. The segregation ratio was 59 male sterile to 63 fertile plants, which agrees with the expected ratio of 50% male sterile (homozygous at *ms8* locus) to 50% fertile (heterozygous) plants.

**Table 1:** Genetic analysis of the sorghum nuclear male sterile mutant *ms8*.

Cross	Number of Plants	F1 Phenotype	Fertile F2	Sterile F2	X^2^ (p-value)
BTX623* *ms8*	8	All Fertile	320	108	0.91
ms8*msx F1	16	9 ms, 7 fertile			0.61
ms1* *ms8* F1	16	All Fertile			
ms2* *ms8* F1	16	All Fertile			
ms3* *ms8* F1	16	All Fertile			
ms7* *ms8* F1	15	All Fertile			
msal* *ms8* F1	13	All Fertile			

The *ms8* male sterility was mediated by a novel recessive mutation in a nuclear gene.

To test if the *ms8* mutant was allelic to other NMS mutants reported previously in sorghum, we obtained all NMS mutants preserved by Dr. Pedersen [16]. These included *ms1*, *ms2*, *ms3*, *ms7*, and *msal*. Pollen collected from the heterozygous *ms8* plants were used to pollinate male sterile plants from the previously reported *ms* mutants. If *ms8* was allelic to those male sterile lines, the progeny would segregate for 50% male sterile and 50% fertile plants. If *ms8* belonged to a different locus from the examined NMS line, all F1 plants would be male fertile. As shown in Table 1, all F1 plants were male fertile for all crosses. Thus, *ms8* represents a new male sterile mutant. We could not determine whether *ms8* was allelic to *ms4*, *ms5*, or *ms6*, because these lines are no longer available. Collectively, our results support that *ms8* is a novel male sterile locus distinct from all NMS lines currently available [21, 22].

### Tapetum and Pollen Development Are Abnormal in *ms8* Anthers

**Fig 4:**
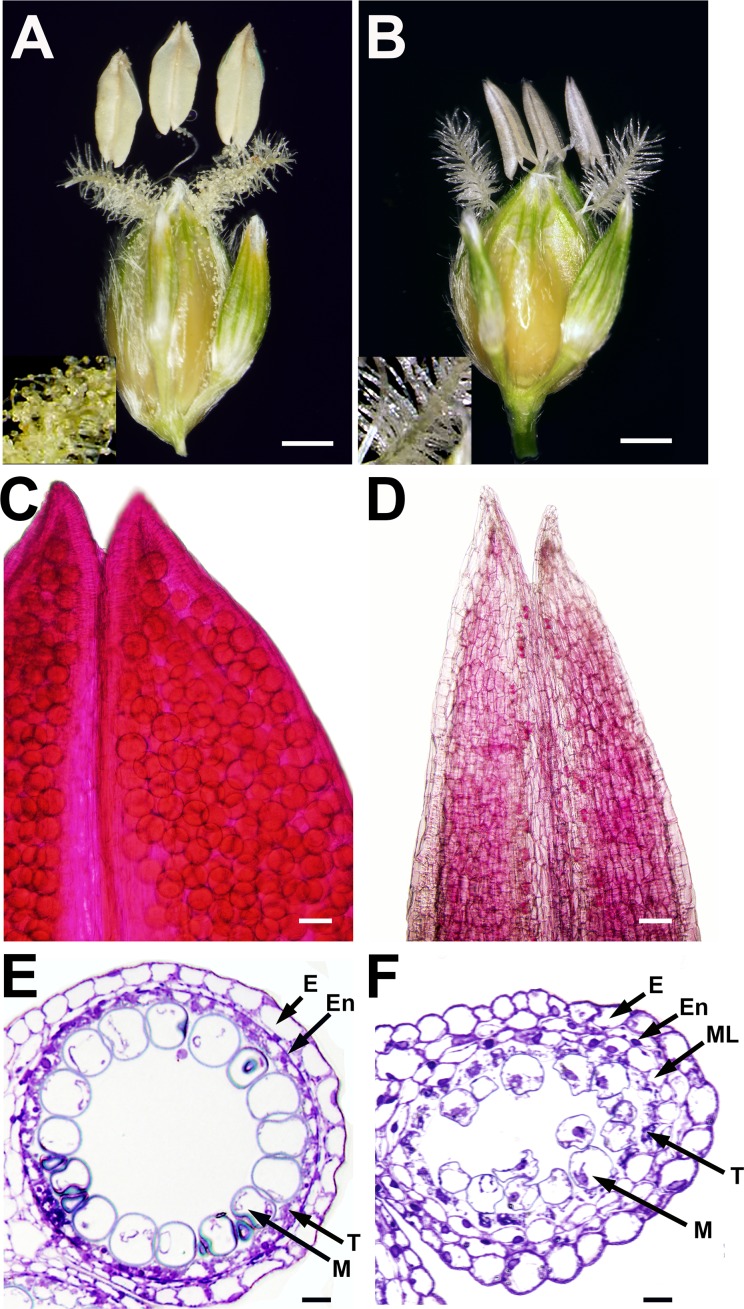
Morphological analyses of pollen and anther development in BTx623 wild-type and *ms8* mutant plants. **(A)** A BTx623 sorghum spikelet with three mature anthers. Pollen grains released from anthers and stacked on hairy stigmas (**A**, inset). **(B)** An *ms8* mutant spikelet with three pale and flattened anthers. No pollen grains on the *ms8* stigma (**B**, inset). **(C)** A part of BTx623 anther showing round pollen grains inside anther lobes. **(D)** A part of *ms8* mutant anther showing no pollen grains inside anther lobes. **(E)** A semi-thin section image of a BTx623 anther lobe at late vacuolated stage showing epidermis, endothecium, tapetum, and microspores. The middle layer (a cell layer between endothecium and tapetum) is degenerated at this stage. **(F)** A semi-thin section image of an *ms8* anther lobe at late vacuolated stage showing epidermis, disordered endothecium and middle layer, early degenerated tapetal cells, and abnormal microspores. E, epidermis; En, endothecium; ML, the middle layer; T, tapetum; M, microspore. Bars = 0.5 mm in **A** and **B**, 20 μm in **C** and **D**, and 10 μm in **E** and **F**.

To investigate what causes male sterility in the *ms8* mutant, we examined pollen viability and anther development. In the BTx623 wild-type mature spikelet (sessile spikelet), there are three extruding yellow anthers and two stigmas with pollen grains (Fig 4A). However, in the *ms8* mutant spikelet (sessile spikelet), three extruding anthers are pale colored and flattened (Fig 4B). In addition, pollen grains were not observed on the *ms8* stigmas (Fig 4B). To evaluate pollen production and viability of *ms8* mutant anthers, we performed Alexander staining of pollen grains prior to anthesis. In BTx623 anthers, round red pollen grains were evenly distributed in anther lobes (Fig 4C). Conversely, no pollen grains were found in *ms8* mutant anthers (Fig 4D). To further examine what results in the failure of pollen production in *ms8* mutant, we carried out semi-thin sectioning of wild-type and *ms8* anthers. In the late-vacuolated stage, except for the degenerated middle layer, the BTx623 anther showed concentrically organized epidermis, endothecium, tapetum, and vacuolated microspores (Fig 4E). However, in the *ms8* mutant anther, abnormal anther cell layers were observed. The tapetum had degenerated prematurely relative to that of BTx623, while the degeneration of the middle layer failed to occur by this stage in the *ms8* mutant (Fig 4F). Moreover, the microspores were collapsing, indicated by their irregular shape (Fig 4F). Our results suggest that the precocious degeneration of tapetum causes abnormal development of microspores, and consequently the absence of pollen grains.

## Discussion

Here we reported a new sorghum NMS mutant, *ms8*, isolated from the mutant library established in sorghum inbred line BTx623 [17]. At present, genetic crosses between two sorghum lines, which is required for breeding and many genetic studies, is primarily carried out by hand emasculation to remove anthers or using plastic bag to prevent pollen shedding from the maternal parent [7]. The hand emasculation method is inefficient and painstaking. The plastic bag method is to tightly wrap a plastic bag around the panicle during anthesis. The resulting high moisture around the panicle prevents anthers from dehiscence. Two or three days after anthesis when pollen grains are died, the anthers are shaken off. After the plastic bag is removed, the panicle is manually pollinated. This method is widely used for making crosses between two lines that are phenotypically different, so that the F1 plants can be visibly separated from the self-pollinated plants because the plastic bag covering cannot kill all pollen grains and the probability to produce self-pollinated is pretty high. The high temperature and high moisture within the plastic bag also kill ovaries, leading to low or no seed set. In addition, the plastic bag method is not feasible for making crosses between two lines that have similar appearance or between mutants isolated from the same genetic background unless molecular markers are available to distinguish the F1 plants from the self-pollinated plants. As an easily recognizable NMS mutant, *ms8* can greatly facilitate breeding and genetic crosses in sorghum.

Because of the ease of recognition at early stage of anthesis, the *ms8* mutant has been used as a convenient tool to backcross other mutants isolated from our sorghum mutant library. The *ms8* mutant may also serve as a valuable tool as an accurate test of heterosis potential during development of new inbred lines in sorghum [23]. Examining heterosis between inbred lines in A/B and R system is complex, time consuming, and expensive. It is often necessary to make crosses with several combinations of A/B and R pairs to determine the value of new inbred lines. The *ms8* mutation can be introduced into a core collection of diverse sorghum accessions that serve as potential B lines, because the *ms8* mutant is derived from the BTx623 B line. The modified B lines with the *ms8* mutation can be used to cross with many diverse R lines to identify desirable levels of heterosis. Once such pairs of lines are identified, large effort can be focused on developing corresponding A/B pair and R lines.

Long-term random mating has been demonstrated as an effective approach for crop improvement in both self-pollination and cross-pollination crops [24, 25]. Sorghum is an essentially self-pollinated species with an outcross rate from 0 to 5% [26]. The *ms8* mutant may aid the development of long-term random mating population for sorghum improvement and genomic selection. Recently, sorghum scientists have empaneled three diversity populations for genome wide association studies on key important agronomic, bioenergy, and nutrition traits in sorghum [1, 27–29]. These diversity panels captured the majority of genomic variations of sorghum and can serve as a powerful initial resource for sorghum improvement through long-term random mating. For example, *ms8* mutant plants can be planted within the field of sorghum diversity panels. Plants homozygous at *ms8* locus can be tagged at anthesis. Because homozygous *ms8* mutants cannot produce any pollen, all seeds on *ms8* plants have to be derived from random mating with pollens from the diversity panel. Only tagged panicles will be harvested and then F2 seeds are produced through self-fertilization. The F2 seeds can be planted into the diversity panel. Again, only the tagged open-pollinated panicles from the F2 plants homozygous at the *ms8* locus will be harvested. This cycle can continue for many generations with or without selection pressure at early stages. Genomic selection or simply breeding selection can be applied at advanced generations to develop sorghum inbred lines that are superior in biotic/abiotic stress resilience, yield, and quality.

Our phenotypic analyses show that the defects in tapetum development result in male sterility in the *ms8* mutant. Tapetum consists of a monolayer or multilayers of cells, which surround successive stages of microsporocytes, tetrads, microspores, and developing pollen as anther development progresses [30–32]. Tapetum development comprises three stages: differentiation, maturation, and programmed cell death (PCD). Early tapetal cells secrete enzymes required for the release of haploid microspores from meiotic tetrads [33–37]. With endoreduplication, polynucleate tapetal cells, which are highly active in metabolism, provide energy and materials (sugar, lipid, and protein) for pollen development. Lacking or abnormal tapetum causes pollen defects and consequently male sterility [18, 19, 38–41]. Moreover, stress-induced male sterility and yield loss are mainly ascribed to aberrant tapetum development [42, 43]. Although some genes important for tapetum development have been identified, so far, the molecular mechanisms underlying tapetal cell differentiation and function maintenance are still not clear. Studies found that dicots (e.g. *Arabidopsis*) and monocots (e.g. maize and rice) employ similar, but also many different genes to control tapetum development [32, 37]. The *ms8* mutant was isolated from the mutant library derived from BTx623 that has been used to generate the sorghum genome sequence [4]. In sorghum, it typically takes several years to isolate a stable male sterile mutant caused by a single gene mutation. To the best of our knowledge, so far, *ms8* is the first nuclear male sterile mutant defective in tapetum development in sorghum. Our future plan is to clone the *MS8* gene via the whole genome sequencing. Considering the moderate large genome and lack of available tools, it is still challenging to identify the *MS8* gene. Once we cloned the *MS8* gene, we will be more aimed to perform detailed morphological, genetic and molecular analyses to examine what cause the mutant phenotype. The *MS8* might be a novel gene in plants or *MS8* orthologs have been studied in other species. In any cases, we are anticipating to clone the first male sterile gene in sorghum, which is potentially important for better understanding of tapetum development and sorghum breeding.

Identification of the *MS8* gene will help develop a two-line hybrid breeding system. For example, an expression cassette of the wild-type *MS8* gene driven by a dexamethasone (DEX) -inducible promoter can be used to transform the *ms8* mutant [44]. Homozygous *ms8* plants can be maintained via treating male sterile plants with DEX. During hybrid production, plants homozygous for the *ms8* mutation will be male sterile in the absence of chemical inducer. The transgene together with the *ms8* mutation can be easily introduced into other sorghum accessions through marker-assisted selection as needed. The advantage of the inducible male fertile system is that we can breed sorghum hybrid under the same condition without the need to produce hybrid seeds and maintain the male sterile line under different conditions.

In summary, we presented a detail morphological characterization of a new and easily recognizable NMS mutant, which was isolated from a sorghum mutant library generated from inbred line BTx623. A male sterile-isogenic line of BTx623 was created by backcrossing *ms8* to BTx623 for 6 generations. Given the easiness to identify the male plants in the field and its stability across different environments, the *ms8* mutant has several important applications in sorghum improvement. First, it can be used to backcross all mutants isolated from the sorghum mutant library, eliminating the need for hand emasculation. Second, it can be a starting parent for sorghum improvement through long-term random mating. Third, it can be introduced in a selection of founder lines for testing heterosis. Fourth, it may eventually lead to the development of an inducible two-line breeding system after the causal gene mutation is identified.
